# Interseason waning of vaccine-induced hemagglutination inhibition antibody titers and contributing factors to pre-existing humoral immunity against influenza in community-dwelling older adults 75 years and older

**DOI:** 10.1186/s12979-023-00362-8

**Published:** 2023-07-31

**Authors:** Bettina Wunderlich, Thomas Laskow, Huifen Li, Li Zhang, Engle Abrams, Jing Tian, Jun Yu, Yiyin Chen, Juliette Tavenier, Yushu Huang, Kawsar Talaat, Jay H. Bream, Qian-Li Xue, Graham Pawelec, Sean X. Leng

**Affiliations:** 1 Department of International Health, Johns Hopkins Bloomberg School of Public Health, Baltimore, MD USA; 2 Center for Immunization Research, Johns Hopkins Bloomberg School of Public Health, Baltimore, MD USA; 3 Division of Geriatric Medicine and Gerontology, Department of Medicine, Johns Hopkins University School of Medicine, Baltimore, MD USA; 4 Division of Geriatric Medicine and Gerontology, Department of Medicine, Johns Hopkins Center On Aging and Immune Remodeling, Johns Hopkins University School of Medicine and Bloomberg School of Public Health, JHAAC Room 1A.38A, 5501 Hopkins Bayview Circle, Baltimore, MD 21224 USA; 5 Department of Geriatrics, The First People’s Hospital of Yunnan Province, Kunming, China; 6 Department of Clinical Research, Copenhagen University Hospital Amager and Hvidovre, Hvidovre, Denmark; 7 Vassar College, Poughkeepsie, NY USA; 8 Department of Molecular Microbiology and Immunology, Johns Hopkins Bloomberg School of Public Health, Baltimore, MD USA; 9 Immunology Training Program, Johns Hopkins School of Medicine, Baltimore, MD USA; 10 Department of Immunology, University of Tübingen, Tübingen, Germany; 11 Health Sciences North Research Institute, Sudbury, ON Canada

**Keywords:** Influenza vaccination, Older adults, HAI antibody titer, Interseason antibody waning, Pre-existing humoral immunity

## Abstract

**Background:**

Seasonal influenza causes significant morbidity and mortality with a disproportionately high disease burden in older adults. Strain-specific hemagglutination-inhibition (HAI) antibody titer is a well-established measure of humoral immunity against influenza and pre-vaccination HAI titer is a valuable indicator of pre-existing humoral immunity at the beginning of each influenza season in highly vaccinated older adults. While vaccine-induced HAI antibody titers are known to wane over time, accurate assessment of their interseason waning has been challenging. This is because pre-vaccination HAI titers are routinely measured using current season vaccine strain antigens instead of the prior season vaccines with which individuals were immunized; as such, they do not accurately represent residual antibody titers from prior season vaccination. This study took advantage of available pre-vaccination HAI titers measured using both current and prior season vaccine strain antigens in a longitudinal influenza immunization study with participants enrolled for multiple consecutive influenza seasons from 2014 through 2017. Influenza A virus (IAV) H3N2 and influenza B virus (IBV) strains in the vaccine formula changed in 2015 and again in 2016 season. IAV H1N1 vaccine strain remained the same from 2014 through 2016 seasons, but changed in 2017. We also investigated factors contributing to pre-existing humoral immunity.

**Results:**

Interseason waning of HAI titers was evident, but rates of waning varied among vaccine strains and study seasons, from 18% (*p* = .43) to 61% (*p* < .01). Rates of waning were noticeably greater when pre-vaccination HAI titers were measured by the routine approach, i.e., using current season vaccine strain antigens, from 33% (*p* = .12) to 83% (*p* < .01), adjusting for age at prior study season, sex, race, and education. This was largely because the routinely measured pre-vaccination HAI titers underrepresented residual HAI titers from prior season vaccinations. Moreover, interseason antibody waning and prior season post-vaccination HAI titers had significant and independent associations with pre-vaccination HAI titers.

**Conclusions:**

The routinely measured pre-vaccination HAI titer overestimates interseason HAI antibody waning as it underestimates residual antibody titers from prior season vaccination when virus strains in the vaccine formula change. Moreover, interseason antibody waning and prior season post-vaccination HAI titers independently contribute to pre-existing humoral immunity in this highly vaccinated, community-dwelling older adult population.

**Supplementary Information:**

The online version contains supplementary material available at 10.1186/s12979-023-00362-8.

## Background

Seasonal influenza causes significant morbidity and mortality each year. The highest burden of severe disease and deaths disproportionally occurs in older adults, particularly those over 75 years of age [1]. Not only does influenza infection cause acute respiratory illness, it can also lead to exacerbation of comorbid chronic conditions, such as cardiovascular diseases in older adults; substantial evidence indicates protective effects of influenza vaccination against both [2–5]. Therefore, annual immunization with influenza vaccines has been recommended for older adults as the primary prevention against this common viral infection for more than 50 years [6, 7].

While correlates of protection of influenza vaccination are not completely understood, humoral immunity is thought to be very important in preventing the infection. This is most commonly assessed by measuring strain-specific hemagglutination-inhibition (HAI) antibody titers. Post-vaccination HAI titers and vaccine-induced HAI antibody responses (i.e., rates of 4-fold or higher rise of post-vaccination titer from pre-vaccination titer or seroconversion) are the main focus of studies of influenza vaccine immunogenicity. However, older adults typically have significant pre-vaccination HAI titers at the beginning of each influenza season, likely from prior annual vaccinations and/or previous influenza infection(s) [8–10]. Substantial evidence suggests that such pre-existing humoral immunity has a major impact on post-vaccination HAI titers and vaccine-induced antibody responses [10–13]. Since vaccine-induced HAI antibody titers are known to wane over time and older adults are a highly vaccinated population [14–18], it is critically important to accurately assess interseason waning of HAI titers from prior season vaccination. This knowledge may also shed light on waning of antibody titers induced by other vaccines, a significant problem common to respiratory viral infections and vaccination including COVID-19 pandemic and immunization with SARS-CoV-2 vaccines [19, 20].

Pre- and post-vaccination HAI titers are routinely measured using current season’s influenza vaccine virus strain antigens. This approach is usually employed to measure intra-season antibody waning from post-vaccination HAI titers. For example, studies have evaluated vaccine immunogenicity, effectiveness, and/or HAI titer waning within a single season [14, 15, 21–24]. However, accurately assessing interseason waning of HAI titers is challenging because the vaccine strain antigens can be different from the prior influenza season. A few studies assessed HAI titers over a long period of time, up to 12-18 months expanding across two seasons, in which the vaccine strain antigens from the index season (i.e., when vaccine was administered) were employed for HAI titer measurement at subsequent time points [25–27]. Two other studies focused on seasons during which the vaccine strain antigens remained the same or simultaneously tested for HAI titers across all strains included in the seasonal vaccines [28, 29]. In a unique clinical setting in which tropical countries may experience more than one influenza season within a single year, Zhao et al employed the same vaccine strain antigens for measuring HAI titers over time across seasons [25].

To determine interseason waning of HAI titers induced by prior season vaccination when influenza virus strains are changed in the vaccine formula, we took advantage of the availability of the pre-vaccination HAI titers that were measured using vaccine strain antigens from both current and prior seasons. From 2014 through 2017 seasons, influenza A virus (IAV) H3N2 and influenza B virus (IBV) strains changed in 2015 and again in the 2016 vaccine formula. While IAV H1N1 vaccine strain remained the same from 2014 through 2016 seasons, it changed in the 2017 season. The data of pre-vaccination HAI titers measured using the corresponding two different vaccine strain antigens and post-vaccination HAI titers measured using the current season vaccine strain antigens were available from Johns Hopkins Longitudinal Influenza Immunization Study of Aging 75 and over (JH LIISA 75+) [11]. JH LIISA 75+ is an ongoing study of annual influenza immunization in community-dwelling older adults 75 years and older with many participants enrolled for multiple consecutive study seasons, making it possible to directly assess interseason waning of HAI titers in the same individuals. The study also provided a unique opportunity for such assessment in a highly vaccinated older adult population with advanced age. In addition, we investigated interseason antibody waning and prior season post-vaccination antibody titer as potentially important factors contributing to pre-existing humoral immunity.

## Results

### Study participants, influenza immunization, and HAI antibody titers

**Fig. 1:**
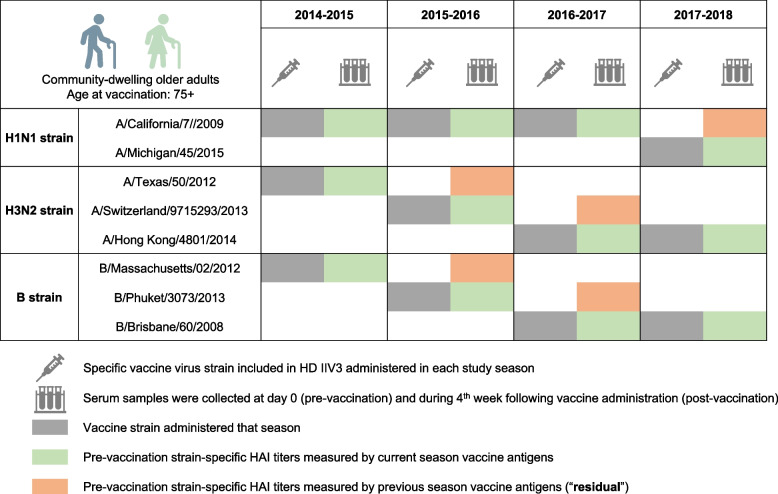
Study design. High dose (HD) inactivated trivalent influenza vaccine (IIV3) administration and specific influenza virus strains included in the vaccine formula (gray horizontal bar) are shown in each study season. Pre-vaccination strain-specific hemagglutination inhibition (HAI) antibody titers were measured using current season vaccine strain antigens (routine approach, green bar) and prior season vaccine strain antigens (orange bar)

Table 1 shows demographic and clinical characteristics of the participants. The mean age of participants during the study was 82-83 years for each of the four study seasons, and there is a preponderance of females (over 50% each year). Over 80% of participants considered themselves white, and the majority had completed college. Over 60% of participants were categorized as either prefrail or frail in each study season based on a validated frailty phenotype [30], with up to 15% categorized as frail in 2015. Figure 1 summarizes the study design and the influenza virus strains included in each season’s vaccine formula. All participants received a high dose trivalent inactivated influenza vaccine (HD IIV3, Fluzone High-Dose, Sanofi, Swiftwater, PA). Post-vaccination titers were measured in serum samples collected four weeks after vaccine administration. To minimize the impact of breakthrough influenza infection on subsequent season pre-vaccination HAI titers and interseason antibody waning, five confirmed influenza cases identified through post-vaccination influenza surveillance (two cases in 2014, one in 2015, and two in 2016) were excluded from this study. For the purpose of the main analyses in this study, participation in two consecutive study seasons was required, yielding a final sample size of 49 for 2014-2015, 52 for 2015-2016, and 78 for 2016-2017 study seasons (additional details can be found in Supplement Table 1).

**Table 1:** Demographic characteristics of the entire study cohort included in this study, by individual study seasons

**Total person-seasons**^a^ **(*n* = 449)**	**2014 (*n* = 74)**	**2015 (*n* = 113)**	**2016 (*n* = 90)**	**2017 (*n* = 172)**
**Age, mean (standard deviation)**	83.3 (5.3)	82.4 (5.6)	82.2 (5.6)	82.8 (5.1)
**Sex (male), n (%)**	30 (39.5)	51 (44.7)	40 (43.5)	69 (40.1)
**Race (white), n (%)**	63 (82.9)	99 (87.6)	79 (85.9)	148 (86.1)
**Education, n (%)**				
**High school**	28 (36.8)	36 (31.9)	32 (34.8)	60 (34.9)
**College**	29 (38.2)	43 (38.0)	41 (44.6)	73 (42.4)
**Graduate school**	19 (25.0)	34 (30.1)	19 (20.6)	39 (22.7)
**Frailty, n (%)**				
**Non-frail**	22 (32.4)	27 (27.0)	32 (38.6)	60 (37.5)
**Prefrail**	40 (58.8)	58 (58.0)	44 (53.0)	80 (50.0)
**Frail**	6 (8.8)	15 (15.0)	7 (8.4)	20 (12.5)

^a^Out of total person-seasons, 113 subjects participated in a single season and 124 subjects participated in at least two consecutive seasons from 2014 through 2017

### Assessment of interseason waning of HAI antibody titers from the immediate prior season vaccination

First, we evaluated interseason HAI titer waning over a period of approximately one year. We compared current season pre-vaccination HAI titer measured with prior season vaccine strain antigens with post-vaccination HAI titer from the immediate prior season for the same individuals, generating a ratio of the two. The rate of interseason HAI titer waning was derived from the formula (1-ratio) x 100%. As shown in Table 2, all ratios were less than 1 with the lowest ratio being 0.39, i.e., the highest rate of waning being 61%. Statistically significant interseason HAI titer waning was observed for IAV-H3N2 between 2015-2016 (58%), IBV strain between 2014-2015 (61%), and IAV-H1N1 between 2016-2017 (35%), all *p*< .01, adjusting for age at prior study season, sex, race, and education. Adjusted rates of interseason waning for IAV-H3N2 between 2014-2015 and IBV between 2015-2016 were 18% (*p*= .43) and 22% (*p*= .13), respectively. Interestingly, the rate of interseason waning for IAV-H3N2 between 2014-2015 seasons was 31% (*p*< .01 by unadjusted analysis) but in adjusted analysis this was non-significantly different at 18%. Similarly, the HAI titer waning ratio for IBV between 2015-2016 seasons was 25% (*p*< .01 in unadjusted analysis) but was not statistically significant in adjusted analysis at 22%. Upon further analysis (Table 3), it was shown that race was likely the driving factor for the IAV-H3N2 difference, where non-whites had an average reduction of 61% and whites saw no change (ratio of 1.00). For IBV, this difference was likely driven by sex, where in adjusted analysis females had a non-significant change of 17% compared to an average of 35% reduction in males.

**Table 2:** Ratios of pre-vaccination HAI titers over prior season post-vaccination HAI titers among participants who completed the study in the corresponding two consecutive seasons when corresponding virus strains in the vaccine formula changed^a^

**Vaccine strain**	**Consecutive two seasons**	**Unadjusted**	**Adjusted**^b^
IAV-H3N2	2014–2015 (*n* = 49)	0.69 (0.53–0.89) (*p* < *.01*)	0.82 (0.51–1.34) (*p* = *.43*)
	2015–2016 (*n* = 52)	0.50 (0.41–0.60) (*p* < *.01*)	0.42 (0.28–0.62) (*p* < *.01*)
IBV	2014–2015 (*n* = 49)	0.37 (0.32–0.42) (*p* < *.01*)	0.39 (0.30–0.50) (*p* < *.01*)
	2015–2016 (*n* = 52)	0.75 (0.64–0.87) (*p* < *.01*)	0.78 (0.56–1.08) (*p* = *.13*)
IAV-H1N1	2016–2017 (*n* = 78)	0.70 (0.60–0.82) (*p* < *.01*)	0.65 (0.49–0.85) (*p* < *.01*)

^a^Pre-vaccination and prior season post-vaccination HAI antibody titers were both measured using the respective prior season vaccine strain antigens

^b^Adjusted for age at prior study season, sex, race, and education

**Table 3:** Effect of race for 2014–2015 IAV-H3N2 and sex for 2015–2016 IBV on unadjusted and adjusted ratios of pre-vaccination HAI titers over prior season post-vaccination HAI titers^a^

**Vaccine strain**	**Consecutive two seasons**	**Unadjusted**	**Adjusted**^b^
IAV-H3N2	2014–2015 white (*n* = 42)	0.70 (0.53–0.93) (*p* < *.012*)	1.00 (0.65–1.53) (*p* = *.9980*)
	2014–2015 non-white (*n* = 7)	0.62 (0.30–1.29) (*p* = *.200*)	0.39 (0.19–0.84) (*p* = *.0154*)
IBV	2015–2016 male (*n* = 20)	0.73 (0.59–0.90) (*p* = *.0038*)	0.65 (0.47–0.90) (*p* = *.0089*)
	2015–2016 female (*n* = 32)	0.76 (0.61–0.94) (*p* = *.0122*)	0.83 (0.52–1.32) (*p* = *.4278*)

^a^Pre-vaccination and prior season post-vaccination HAI antibody titers were both measured using the respective prior season vaccine strain antigens

^b^Adjusted for age at prior study season, sex, race, and education

### Associations of interseason antibody waning with current season pre-vaccination HAI antibody titers

**Fig. 2:**
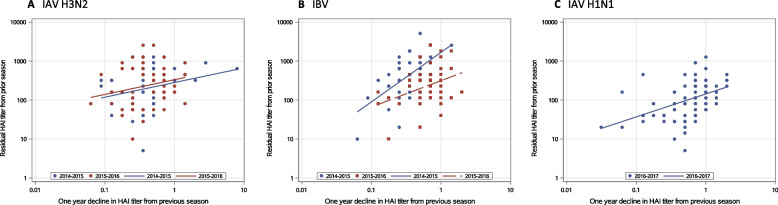
Relationships between current season pre-vaccination HAI antibody titers and interseason antibody decline (waning). Associations of current season pre-vaccination HAI antibody titers measured using prior season vaccine strain antigens with interseason antibody decline (waning) as indicated by the ratios shown in Table 2 were evaluated in univariate analyses. **A** IAV-H3N2: Spearman correlation coefficient between 2014–2015 (*n* = 49) was 0.30 (*p* = .04) and that between 2015–2016 (*n* = 52) was 0.23 (*p* = .10); **B** IBV: Spearman correlation coefficient between 2014–2015 (*n* = 49) was 0.49 (*p* < .01) and that between 2015–2016 (*n* = 52) was 0.34, (*p* = .02); **C** IAV-H1N1: Spearman correlation coefficient between 2016–2017 (*n* = 78) was 0.51 (*p* < .01)

Because pre-vaccination HAI titer is an important indicator of pre-existing humoral immunity against influenza in older adults at each season, we investigated which factors contributed to pre-vaccination HAI titers in this highly vaccinated older adult population. We began with an exploratory analysis of the relationships between interseason antibody waning and pre-vaccination HAI titers. In this analysis, the ratio of pre-vaccination titer to prior season post-vaccination titer shown in Table 2 was employed as the indicator of interseason antibody waning. Figure 2 illustrates the relationships obtained from univariate analysis between current season pre-vaccination HAI titers, also referred to as “residual”, and interseason antibody decline, or waning. Of note, a higher ratio (all ratios are less than 1 as shown in Table 2) is an indicator of less interseason waning. As such, a positive association with a significant Spearman correlation coefficient indicates an inverse relationship. That is, a lower interseason HAI titer waning is associated with a higher pre-vaccination HAI titer. This analysis yielded statistically significant associations between pre-vaccination HAI titers and interseason antibody waning for all vaccine strains and study seasons except for IAV-H3N2 between 2015-2016 (*ρ*=0.23, *p*= .10) (Fig. 2A-C).

Consistent with these results from univariate analysis, except for IAV-H3N2 between 2014-2015 or between 2015-2016 (data not shown), estimated effects of interseason antibody waning on pre-vaccination HAI titers were statistically significant for IBV between 2014-2015 and between 2015-2016 (1.34 [1.13-1.59], *p*< .01 and 1.10 [1.02-1.19], *p*= .02, respectively) and IAV-H1N1 between 2016-2017 (1.11 [1.05-1.17], *p*< .01), all adjusted for age at prior study season, sex, race, and education.

### Associations of prior season post-vaccination HAI antibody titers with current season pre-vaccination HAI titers

**Fig. 3:**
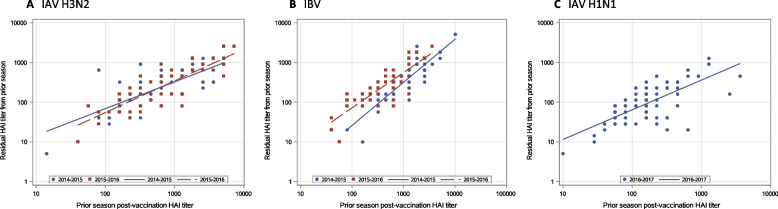
Relationships between current season pre-vaccination HAI titers (residual) and prior season post-vaccination HAI antibody titers. Associations of pre-vaccination HAI antibody titers with prior season post-vaccination HAI titers were evaluated in univariate analyses. **A** IAV-H3N2: Spearman correlation coefficient between 2014–2015 (*n* = 49) was 0.72 and that between 2015–2016 (*n* = 52) was 0.82, *p* < .01 for both; **B** IBV: Spearman correlation coefficient for between 2014–2015 (*n* = 49) was 0.89 and that between 2015–2016 (*n* = 52) was 0.80, *p* < .01 for both; **C** IAV-H1N1: Spearman correlation coefficient between 2016–2017 (*n* = 78) was 0.66, *p* < .01

We also evaluated relationships between prior season post-vaccination HAI titers and current season pre-vaccination HAI titers in a univariate analysis (Fig. 3). The results indicate significant associations across all vaccine strains and study seasons, with the strongest associations seen for IBV (*ρ*=0.89 in 2014-2015) (Fig. 3B).

Consistent with these results from univariate analysis, estimated effects of prior season post-vaccination HAI titers on pre-vaccination HAI titers were statistically significant for IAV-H3N2 between 2014-2015 and between 2015-2016 (1.52 [1.33-1.74] and 1.72 [1.55-1.90], respectively, both *p*< .01), IBV between 2014-2015 and between 2015-2016 (2.16 [1.92-2.43] and 1.83 [1.65-2.02], respectively, both *p*< .01), and IAV-H1N1 between 2016-2017 (1.82 [1.61-2.04], *p*< .01), all adjusted for age at prior study season, sex, race and education. In fact, the effect size for IBV between 2014-2015 was the largest, exhibiting a two-fold increase in prior season post-vaccination HAI titer associated with a 116% increase in current season pre-vaccination HAI titer (*p*< .01).

### Independent effects of interseason antibody waning and prior season post-vaccination HAI antibody titer on current season pre-vaccination HAI titers

We further explored independent effects of interseason antibody waning and prior season post-vaccination HAI titers on pre-vaccination HAI titers, as summarized in Table 4. In Model 1, significant effects of interseason HAI antibody waning were retained after adjusting against prior season post-vaccination HAI titers, with or without adjusting for additional covariates (i.e., age at prior study season, sex, race, and education), for all vaccine strains and study seasons. In Model 2, significant effects of prior season post-vaccination titers were retained after adjusting against interseason antibody waning, with or without adjusting for additional covariates, for all vaccine strains and study seasons.

**Table 4:** Independent effects of interseason antibody waning and prior season post-vaccination HAI antibody titers on current season pre-vaccination HAI titers^a^

		**Model 1**^b^ **Interseason titer waning**		**Model 2**^c^** Prior season post-vaccination titers**	
**Vaccine Strain**	**Consecutive two seasons**	**Adjusted for prior season post-vaccination titers**	**Adjusted for additional covariates**^c^	**Adjusted for interseason antibody waning**	**Adjusted for additional covariates**^c^
IAV-H3N2	2014–2015 (*n* = 49)	1.06 (1.04–1.08) (*p* < .01)	1.06 (1.04–1.08)(*p* < .01)	1.82 (1.65–1.99) (*p* < .01)	1.83 (1.67–2.01) (*p* < .01)
	2015–2016 (*n* = 52)	1.21 (1.18–1.24) (*p* < .01)	1.21 (1.18–1.24) (*p* < .01)	1.98 (1.90–2.07) (*p* < .01)	1.98 (1.90–2.07) (*p* < .01)
IBV	2014–2015 (*n* = 49)	1.27 (1.22–1.31) (*p* < .01)	1.26 (1.22–1.31) (*p* < .01)	2.04 (1.94–2.13) (*p* < .01)	2.04 (1.95–2.14) (*p* < .01)
	2015–2016 (*n* = 52)	1.16 (1.14–1.18) (*p* < .01)	1.16 (1.14–1.18) (*p* < .01)	2.02 (1.94–2.10) (*p* < .01)	2.02 (1.94–2.11) (*p* < .01)
IAV-H1N1	2016–2017 (*n* = 78)	1.17 (1.15–1.19) (*p* < .01)	1.17 (1.14–1.19) (*p* < .01)	1.91 (1.81–2.00) (*p* < .01)	1.92 (1.82–2.03) (*p* < .01)

^a^Summary of estimated effects of interseason antibody waning adjusting for prior season post-vaccination HAI antibody titers (Model 1) and vice versa (Model 2) on current season pre-vaccination HAI antibody titers. Notes: Outcome measure is current season pre-vaccination HAI antibody titers measured using prior season vaccine strain antigens

^b^Estimated effects are expressed as expected fold-change (95% confidence interval) in current season pre-vaccination HAI titers that is associated with a 0.1-unit less interseason antibody waning after adjusting against prior season post-vaccination HAI antibody titers (Model 1) or a two-fold increase in prior season post vaccination HAI antibody titers after adjusting against interseason antibody waning (Model 2), with or without adjusting for additional covariates (both Model 1 and 2)

^c^Additional covariates include age at prior study season, sex, race, and education

### Interseason waning of HAI antibody titers when assessed using current season vaccine strain antigens

We also evaluated interseason HAI antibody titer waning when the current season pre-vaccination HAI titers were determined using current season vaccine strain antigens. As shown in Table 5, all ratios were less than 1 with the lowest one being 0.17 indicating a waning rate of 83%. Except for IBV strain for the 2015-2016 season (33%, *p*= .12), interseason antibody waning for all other vaccine strains and study seasons were between 41% and 83% with statistical significance (*p*< .01) after adjusting for age at prior study season, sex, race, and education.

**Table 5:** Interseason antibody waning estimated using current season vaccine strain antigens^a^

**Vaccine strain**	**Consecutive two seasons**	**Unadjusted**	**Adjusted**^b^
IAV-H3N2	2014–2015 (*n* = 49)	0.27 (0.20–0.36) (*p* < *.01*)	0.25 (0.14–0.44) (*p* < *.01*)
	2015–2016 (*n* = 52)	0.25 (0.20–0.33) (*p* < *.01*)	0.21 (0.12–0.35) (*p* < *.01*)
IBV	2014–2015 (*n* = 49)	0.18 (0.15–0.22) (*p* < *.01*)	0.17 (0.12–0.24) (*p* < *.01*)
	2015–2016 (*n* = 52)	0.96 (0.74–1.24) (*p* = *.74*)	0.67 (0.40–1.12) (*p* = *.12*)
IAV-H1N1	2016–2017 (*n* = 78)	0.58 (0.50–0.68) (*p* < *.01*)	0.59 (0.45–0.77) (*p* < *.01*)

^a^Ratios of pre-vaccination HAI antibody titers measured using current season vaccine strain antigens over prior season post-vaccination HAI titers measured using prior season vaccine strain antigens among participants who completed the study in the two consecutive study seasons

^b^Adjusted for age at prior study season, sex, race, and education

### Comparison between pre-vaccination HAI antibody titers measured using current season vaccine strain antigens and those measured by prior season vaccine strain antigens

We then compared pre-vaccination HAI titers measured using current season vaccine strain antigens over those measured using prior season vaccine strain antigens. As shown in Table 6, pre-vaccination HAI titers measured using current season vaccine strain antigens, which is routinely performed, were lower than those measured using prior season vaccine strain antigens. Specifically, ratios of routinely measured pre-vaccination HAI titers over those measured using prior season vaccine strain antigens for IAV H1N1 strain in 2017 season, H3N2 in 2015 and 2016, and IBV in 2015 were 0.86, 0.36, 0.56 and 0.41 respectively, all statistically significant (*p*= .04, *p*< .01, *p*= .03, and *p*< .01 respectively), adjusting for age at prior study season, sex, race, and education. Such a ratio for IBV strain in 2016 season was 0.84 and did not reach statistical significance (*p*= *.55*). These results were confirmed when we conducted the same analysis in a larger sample size consisting of all participants with both sets of pre-vaccination HAI titers data available in any individual study season (excluding 5 breakthrough influenza cases) (Supplement Table 2).

**Table 6:** Ratios of pre-vaccination HAI antibody titers measured using current season vaccine strain antigens over those using prior season vaccine strain antigens among individuals who participated in respective two consecutive study seasons^a^

**Vaccine strain**	**Individual study season**	**Unadjusted**	**Adjusted**^b^
IAV H3N2	2015 (*n* = 49)	0.39 (0.32–0.47) (*p* < *.01*)	0.36 (0.36–0.50) (*p* < *.01*)
	2016 (*n* = 52)	0.64 (0.50–0.81) (*p* < *.01*)	0.56 (0.33–0.94) (*p* = *.03*)
IBV	2015 (*n* = 49)	0.48 (0.41–0.55) (*p* < *.01*)	0.41 (0.32–0.51) (*p* < *.01*)
	2016 (*n* = 52)	1.77 (1.32–2.37) (*p* < *.01*)	0.84 (0.47–1.49) (*p* = *.55*)
IAV H1N1	2017 (*n* = 78)	0.86 (0.79–0.94) (*p* < *.01*)	0.86 (0.75–0.99) (*p* = *.04*)

^a^Ratios of pre-vaccination HAI titers measured using current study season vaccine strain antigens over those measured using prior season vaccine strain antigens among individuals who participated in the same two consecutive study seasons as the main analysis

^b^Adjusted for age at the study season, sex, race, and education

## Discussion

In this study, the longitudinal nature of the JH LIISA 75 + study cohort with participants enrolled for multiple consecutive influenza seasons has enabled us to directly assess interseason antibody waning and explore factors that contribute to pre-existing humoral immunity in community-dwelling older adults 75 years and older. This was made possible by the availability of data on both current season pre-vaccination and prior season post-vaccination HAI titers measured using the prior season vaccine strain antigens when the corresponding vaccine strains in the vaccine formula changed between the two consecutive study seasons. Our results demonstrate that while interseason antibody waning is evident, the rates of such waning are highly variable depending on vaccine strains and influenza seasons. Moreover, we have identified both interseason antibody waning and prior season post-vaccination HAI titers as major factors that impact pre-existing humoral immunity in this highly vaccinated older adult population.

Intra-(within) season waning of strain-specific HAI titers has been recognized since the first successful trial of inactivated influenza vaccine in 1943 in which Francis and colleagues reported that vaccine-induced HAI antibody titers waned by about one-third 4–5 months after vaccination [31]. However, studies have also showed that vaccine-induced HAI titers could be maintained at high levels in persons ≥ 60 years of age for at least 4 months (summarized in [14]). While interseason antibody waning is likely, its accurate assessment has been challenging. This is because pre-vaccination HAI titers are routinely measured using current season vaccine strain antigens in clinical and influenza vaccine research settings. Such routinely measured pre-vaccination HAI titers do not accurately represent “residual” antibody titers from prior season vaccination when virus strains in the vaccine formula change. Whether this would lead to overestimating interseason antibody waning had not been determined. Here, we took the advantage of available pre-vaccination HAI titers measured using current season vaccine strain antigens (the routine approach) and prior season vaccine strain antigens and directly addressed this issue. Our results support the notion that the routinely measured pre-vaccination HAI titers using current season vaccine strain antigens overestimate the rate of interseason antibody waning; the explanation for such overestimation is its inaccurate assessment of residual HAI antibody titers from prior season vaccination. An exception is the IBV strain of Victoria lineage in the 2016 season as the routinely measured pre-vaccination HAI titers against this vaccine strain were almost two-fold higher than the residual antibody titers measured using prior season IBV strain antigens, as shown in Table 6 and confirmed in a larger sample size for each study season shown in Supplement Table 2. The reason for this exception is not entirely clear, but may be related to the fact that IBV strains are highly conserved and the repeated exposure to similar strains over the years has instilled robust humoral immunity in older adults [32, 33]. It may also be related to an unusually high cross reactivity of pre-vaccination HAI antibody against the 2016 vaccine IBV strain. As such, the interseason waning of HAI titers against IBV between 2015–2016 was not statistically significant, particularly when pre-vaccination HAI antibody titers were measured using current season vaccine strain antigens (Table 5). While interseason HAI titer waning was evident for the majority of participants over one year, 5 to 7 participants had a higher HAI titer than the year before (ratios of > 1, Fig. 2). This interesting finding, if confirmed in other studies, deserves further investigation because all confirmed cases of breakthrough influenza infection were excluded from this study.

Because of the clinical and immunological importance of pre-existing humoral immunity characterized by pre-vaccination HAI antibody titers, we sought to identify factors contributing to pre-vaccination HAI titers. One accepted contributing factor is interseason antibody waning. Our results indicate that less interseason antibody waning is associated with higher current season pre-vaccination HAI titers. However, such an association was not consistent across all vaccine strains or study seasons evaluated. Our study participants are drawn from a highly vaccinated older adult population with repeated annual vaccinations throughout many prior influenza seasons. In this context, it is likely that contribution of interseason antibody waning from any particular season vaccination, e.g., the immediate prior season in this case, to the pre-vaccination HAI titers, may represent a portion of the likely accumulative effect and can be impacted by other covariates and, therefore, may vary. On the other hand, the prior season post-vaccination HAI titers appear to be a strong and consistent contributing factor across all vaccine strains and seasons studied. Theoretically, a high post-vaccination antibody titer in the prior season represents a high starting point for interseason antibody waning, leading to a high residual antibody titer for the next influenza season. Results from further analyses indicate independent effects of both interseason antibody waning and prior season post-vaccination HAI titers, highlighting the importance of both. Nevertheless, further longitudinal studies are warranted to determine whether a high prior season post-vaccination HAI titer and/or a low rate of interseason antibody waning serve as a predictor for a robust pre-existing humoral immunity and, therefore, provide better clinical protection against influenza for older adults. However, it is important to note that the observed associations may not be due to prior season post-vaccination HAI titers or interseason antibody waning rate per se. Instead, these associations may reflect participants experiencing different levels of age-related senescent remodeling of the immune system, or immunosenescence. For example, those with high prior season post-vaccination HAI titers and/or low rates of interseason antibody might have experienced less immunosenescent remodeling even in their advanced age and, therefore, could mount a more robust HAI antibody responses to influenza vaccination each season. It is conceivable that these parameters could be used as an indicator of certain unmeasured intrinsic immune system characteristics, which might include the maintenance of memory B cells and plasma cells for low-level antibody production to counteract antibody waning and provide for further understanding of influenza immunity to identify vulnerable populations who are unable to mount robust humoral immune responses to vaccination. We also found that sex had an impact on waning, where in adjusted analysis females were shown to have less waning of IBV titers in 2015–2016. This is likely explained by the fact that females have been found to develop higher antibody responses to influenza vaccination [34]. For IAV H3N2 2014–2015, race was found to be associated with different rates of waning, where those who identified as non-white had higher rate of waning than those who identified as white. This is interesting because a study by Kurupati et al. found that African Americans mounted higher influenza antibody responses compared to Caucasians [35]. However, other races might show less robust antibody response, and the sample size of non-white (7) was much smaller than those who identified as white (42). We did not find that frailty had significant impact on interseason HAI titer waning in this study likely due to a small sample size. While our previous study showed significant association of the frailty phenotype with lower vaccine-induced HAI antibody titer response and poor clinical protection from a standard dose trivalent inactivated influenza vaccine (SD IIV3) in a similar geriatric population [36], our recent work did not find any significant impact of frailty on pre-vaccination HAI titers in the JH LIISA 75 + study cohort [11].

The strengths of this study include: 1) a well characterized population of community-dwelling older adults 75 years and older with participants enrolled for multiple consecutive influenza seasons; 2) a unique opportunity of available pre-vaccination HAI titers measured using both current season and prior season vaccine strain antigens for in-depth characterization of interseason antibody waning and contributing factors to pre-existing humoral immunity; and 3) ability to exclude breakthrough influenza cases identified through vigorous post-vaccination influenza surveillance to minimize their impact on pre-existing humoral immunity in the following season and interseason antibody waning.

The study also has limitations. For example, our data do not address vaccine effectiveness, a direct and important clinical outcome measure that would require a large sample size. However, strain-specific HAI antibodies are considered to be the major immune mechanism mediating vaccine-induced protection against influenza infection [37] and serve as the basis of age-specific immunogenicity criteria employed by the regulatory committees of the European Medicines Agency (EMA) and the US Federal Drug Administration (FDA) for the approval of new influenza vaccines [38]. Another limitation is the relatively small sample size. While JH LIISA 75 + cohort enrolled significant number of participants in each study season, the sample size for this study is limited by the requirement for participation in two consecutive study seasons. To partially address this limitation, the analysis of comparisons of two sets of pre-vaccination HAI titers presented in Table 6 was repeated and validated among all participants who completed individual study seasons with a larger sample size (Supplement Table 2). In addition, all participants received the high-dose inactivated influenza vaccine (HD-IIV3). Thus, we were not able to compare interseason HAI titer waning against other influenza vaccines recommended to older adults, such as the adjuvanted inactivated influenza vaccine (aIIV3). As there are conflicting results comparing the effectiveness between HD-IIV3 and aIIV3 for older adults [39, 40], it would be desirable to conduct a similar analysis with a longitudinal cohort in which significant number of participants receive either of the two vaccines. Finally, given the nature of the hemagglutination inhibition assay, while pre-vaccination HAI antibody titers measured using prior season vaccine strain antigens are more likely representative of residual antibodies from prior season vaccination, “cross-reactivity” cannot be completely excluded, i.e., such measurements may also indicate HAI antibodies cross-reactive to viral strain antigens from prior influenza vaccinations and/or breakthrough infections accumulated over many previous influenza seasons. Despite these limitations, findings from this study provide a framework for considering a more accurate assessment of residual antibodies from prior season vaccination, interseason antibody waning, and factors contributing to pre-existing humoral immunity in this highly vaccinated older adult population. They also provide initial evidence suggesting that the extent of interseason HAI antibody waning is not as pronounced as indicated when pre-vaccination HAI titers are routinely measured using current season vaccine strain antigens in this subset of older adults. To further address intra- and interseason waning of vaccine-induced humoral immunity as well as pre-existing humoral immunity in older adults, more in-depth investigations including detailed intra-seasonal HAI antibody analyses with frequent sampling after vaccine administration until the beginning of the next influenza season in longitudinal studies as well as vaccine clinical effectiveness are indicated.

## Conclusions

Results from this study demonstrate that while interseason strain-specific HAI antibody waning is evident in older adults, the extent of such waning is not as pronounced as suggested when pre-vaccination HAI titers are routinely measured using current season vaccine strain antigens in community-dwelling older adults 75 years and older. In addition, the rate of interseason antibody waning and prior season post-vaccination antibody titers are major factors that independently contribute to pre-existing humoral immunity in this highly vaccinated older adult subpopulation.

## Methods

### The study population and protocol

JH LIISA 75 + is a prospective observational study of influenza immunization in community-dwelling older adults 75 years and older. The study was started in 2014 and is currently ongoing. Subjects were recruited via collaborating physicians, community newspaper advertisement and flyers at outpatient clinics, senior centers, and residential areas in Baltimore, Maryland. Candidates who consented to participate were screened by trained clinical research coordinators. Exclusion criteria include a history of allergic reaction to influenza vaccine or egg, currently on oral steroids or immunosuppressive therapy, worsening or new-onset of immune-modulating conditions (e.g., rheumatoid arthritis, hematologic malignancies, etc.), or acute illness such as a viral infection. In each of the study seasons, screening and pre-vaccination evaluation were started in late summer, 4–6 weeks before annual massive influenza vaccination in the Baltimore area. Study participants came to the Clinical Research Unit at Johns Hopkins Institute of Clinical and Translational Research or Biology of Healthy Aging Studies Unit on the Johns Hopkins Bayview Medical Center campus, or study visits were conducted at participants’ homes as needed. Detailed demographic and clinical information were obtained. After a pre-vaccination blood draw, participants received a high dose trivalent inactivated influenza vaccine (HD-IIV3, Fluzone® High-Dose, Sanofi, Swiftwater, PA), one of the FDA-approved influenza vaccines specifically recommended for older adults. A second (post-vaccination) blood sample was collected during the 4^th^ week following vaccine administration, and then, post-vaccination influenza surveillance was conducted in each study season until the end of April. From 2014 to 2017, a total of 237 participants were enrolled in JH LIISA 75 + cohort. Among them, 113 only participated in one study season and 124 participated in at least two seasons. For individual study seasons, there were 74 unique subjects for 2014, 114 for 2015, 92 for 2016 and 172 for 2017, leading to a total sample of 454 season-persons for the entire four-season study period (Supplement Table 1).

### Influenza virus strains in the vaccine formula for 2014 through 2017 study seasons

From 2014 through 2017, HD-IIV3 for each season contains 60 μg of hemagglutinin (HA) antigen for each of the three influenza virus strains, namely, IAV H1N1 and H3N2 plus IBV. IAV H1N1 vaccine strain remained the same from 2014 through 2016, A/California/07/2009, but changed to A/Michigan/45/2015 X-275 (pdm-09-like) in 2017. However, IAV H3N2 and IBV vaccine strains were different for each of the 2014, 2015, and 2016 seasons, namely, A/Texas/50/2012, A/Switzerland/9715293/2013, and A/Hong Kong/4801/2014 for 2014, 2015, and 2016, respectively; B/Massachusetts/02/2012 (Yamagata lineage), B/Phuket/3073/2013 ether-treated (Yamagata lineage), and B/Brisbane/60/2008 ether-treated (Victoria lineage) for 2014, 2015, and 2016, respectively (Fig. 1).

### Measurement of strain-specific anti-influenza HAI antibody titers

A validated HAI assay was used to quantify antibody titers against study vaccine antigens for the three vaccine strains (H1N1, H3N2 and B) in each year and were performed by Sanofi as previously described [11, 41]. Briefly, serum was incubated with type III neuraminidase to eliminate non-specific inhibitors and then with turkey red blood cells to adsorb non-specific agglutinins. Two-fold serial dilutions of sera, beginning at a 1:10 dilution, were then performed in duplicate, and sera were incubated with influenza virus (4 hemagglutination units/25 μl). Turkey red blood cells were then added, and the titer defined as the highest dilution in which hemagglutination of turkey red blood cells was inhibited.

### Data analysis

Baseline demographics including age, sex, race, and education were compared by influenza season between 2014 and 2017. To analyze inter-season waning in HAI antibody titers, we modeled the ratio of current season pre-vaccination HAI antibody titers (Y_current_) over prior season post-vaccination HAI titers (Y_prior_) using the generalized linear model (GLM) with a log link and a Gamma error distribution, i.e., log[E(Y_current_/Y_prior_)] = β0 + β1Z, where E(Y_current_/Y_prior_) is the mean value of the ratio; and Z represents a vector of confounders. Exp(β1) therefore can be interpreted as the mean ratio of Y_current_/Y_prior_ after controlling for covariates Z (i.e., mean age, female sex, white race, and high school or below education). Crude and covariate-adjusted analyses were conducted separately for each influenza vaccine strain (i.e., IAV H1N1 and H3N2 and IBV). We also examined the differences in the estimates of the ratio when current season pre-vaccination HAI antibody titers were measured by current season vaccine strain antigen (routine approach) versus those measured by the vaccine strain antigens with which the same participant was immunized in the prior season. To directly assess the differences in the measurement of current season HAI antibody titers by using current season versus prior season vaccine strain antigens, we also used GLMs to estimate the crude and adjusted ratio of current season pre-vaccination HAI antibody titers measured by the two types of vaccine strain antigens. Next, we analyzed the crude and adjusted associations of current-season pre-vaccination HAI antibody titers measured using previous season vaccine strain antigens with both (separately and jointly) the interseason waning in HAI antibody titers and prior-season post vaccination HAI antibody titers. In this model, the interseason waning was defined as the ratio of current season pre-vaccination HAI antibody titer over prior-season post vaccination HAI antibody titer. The effect size from this model was expressed as expected fold change in the current-season pre-vaccination HAI antibody titers that is associated with a 0.1 unit increase in the ratio (or two-fold increase in prior-season post vaccination HAI antibody titers). We similarly explored the impact on the associations when the current season HAI antibody titers were measured using the routine approach. All analyses were performed using Stata version 15.

## Supplementary Information

**Additional file 1: Supplement Table 1.** Subjects’ participation patterns among four study seasons. **Supplement Table 2.** Ratios of pre-vaccination HAI antibody titers measured using current season vaccine strain antigens over those using prior season vaccine strain antigens among all participants in the corresponding individual study seasons^a^.

## Data Availability

The datasets used and/or analyzed during the current study are available from the corresponding author on reasonable request.
