# Supplementary material for: Interseason waning of vaccine-induced hemagglutination inhibition antibody titers and contributing factors to pre-existing humoral immunity against influenza in community-dwelling older adults 75 years and older
Source: Immun Ageing. 2023 Jul 31;20:38. doi: 10.1186/s12979-023-00362-8 (PMC10388475; doi:10.1186/s12979-023-00362-8)
Supplement: Supplementary file 1 — Additional file 1: Supplement Table 1. Subjects’ participation patterns among four study seasons. Supplement Table 2. Ratios of pre-vaccination HAI antibody titers measured using current season vaccine strain antigens over those using prior season vaccine strain antigens among all participants in the corresponding individual study seasonsa. [file 12979_2023_362_MOESM1_ESM.docx]

**Supplement Materials**

**Supplement Table 1. Subjects’ participation patterns among four study seasons.**

| **Number of seasons participated** | **Pattern of participation** | **Frequency**  **(Total n=237)** | **Percent**  **(%)** |  |
| --- | --- | --- | --- | --- |
|  |  |  |  |  |
| 1 | 2014 season only | 18 | 7.59 |  |
|  | 2015 season only | 26 | 10.97 |  |
|  | 2016 season only | 8 | 3.38 |  |
|  | 2017 season only | 61 | 25.74 |  |
| 2 | 2014 and 2015 seasons only | 9 | 3.8 |  |
|  | 2014 and 2017 seasons only | 5 | 2.11 |  |
|  | 2015 and 2016 seasons only | 2 | 0.84 |  |
|  | 2015 and 2017 seasons only | 16 | 6.75 |  |
|  | 2016 and 2017 seasons only | 28 | 11.81 |  |
| 3 | 2014, 2015, and 2016 seasons only | 2 | 0.84 |  |
|  | 2014, 2015, and 2017 seasons only | 10 | 4.22 |  |
|  | 2014, 2016, and 2017 only | 3 | 1.27 |  |
|  | 2015, 2016, and 2017 seasons only | 20 | 8.44 |  |
| 4 | All 4 seasons | 29 | 12.24 |  |

**Supplement Table 2.** Ratios of pre-vaccination HAI antibody titers measured using current season vaccine strain antigens over those using prior season vaccine strain antigens among all participants in the corresponding individual study seasons^a^

| **Vaccine strain** | **Individual study season** | **Unadjusted** | **Adjusted^b^** |
| --- | --- | --- | --- |
| IAV-H3N2 | 2015 (n=114) | 0.41 (0.36-0.47) (*p< .01*) | 0.37 (0.30-0.46) (*p< .01*) |
|  | 2016 (n=91) | 0.66 (0.55-0.79) (*p< .01*) | 0.69 (0.50-0.93) (*p= .02*) |
| IBV | 2015 (n=114) | 0.47 (0.43-0.52) (*p< .01*) | 0.41 (0.35-0.48) (*p< .01*) |
|  | 2016 (n=91) | 2.26 (1.78-2.87) (*p< .01*) | 2.38 (1.61-3.52) (*p< .01*) |
| IAV-H1N1 | 2017 (n=170) | 0.86 (0.82-0.91) (*p< .01*) | 0.85 (0.77-0.93) (*p< .01*) |

^a^The same analysis as in Table 6 except that the sample size included all participants in each specified study season excluding influenza cases identified through post-vaccination influenza surveillance in the respective prior study season. ^b^Adjusted for age at the study season, sex, race, and education.
